# From Surgery to Endoscopy: Comprehensive Review of Bariatric Approaches in Pediatric and Adolescent Patients

**DOI:** 10.3390/medicina62030463

**Published:** 2026-02-28

**Authors:** Carola D’Agostino, Michela Orsi, Alain Garcia Vázquez, Silvana Perretta, Elisa Reitano

**Affiliations:** 1 IHU, Institute of Image-Guided Surgery, 1 Pl. de l’Hôpital, 67000 Strasbourg, France; carola.dagostino@unimi.it (C.D.);; 2 Department of Pediatric Surgery, Fondazione IRCCS Ca’ Granda Ospedale Maggiore Policlinico, 20122 Milan, Italy; 3 Department of Surgical Sciences, University of Rome Tor Vergata, 00133 Rome, Italy; 4 Department of General Surgery, Nouvel Hôpital Civil, University of Strasbourg, 1 pl. de l’Hôpital, 67000 Strasbourg, France

**Keywords:** pediatric obesity, adolescent obesity, bariatric surgery, bariatric endoscopy, medication

## Abstract

*Background and Objectives*: Pediatric and adolescent obesity is a growing global health concern that is often associated with cardiometabolic comorbidities. Lifestyle interventions represent first-line therapy; however, many adolescents with moderate-to-severe obesity fail to achieve clinically meaningful weight loss. The objective of this review is to provide a comprehensive overview of surgical and endoscopic interventions for adolescent obesity. *Materials and Methods*: A structured search of PubMed, Scopus, Web of Science and the Cochrane Library was conducted. Studies reporting outcomes of bariatric surgery (sleeve gastrectomy (SG), Roux-en-Y gastric bypass (RYGB), adjustable gastric banding (AGB)) and endoscopic interventions (endoscopic sleeve gastroplasty (ESG) and intragastric balloons (IGBs)) in patients ≤ 21 years were included. Data on weight loss, BMI reduction, metabolic outcomes, adverse events and follow-up were extracted and summarized. *Results*: Bariatric surgery remains the most effective intervention, with SG and RYGB achieving substantial and durable weight loss and high rates of comorbidity remission. ESG is an emerging intervention with preliminary short-term efficacy and safety data, though evidence is limited to small pediatric cohorts. IGBs provide reversible, non-surgical weight reduction with consistent short-term efficacy, but long-term durability is variable and largely dependent on adherence to behavioral programs. Across all interventions, psychosocial support, family involvement and multidisciplinary care significantly influence the outcomes. *Conclusions*: Procedural interventions play a pivotal role in adolescents with moderate-to-severe obesity. IGBs could represent a minimally invasive, reversible option, particularly as bridging or adjunctive therapy. Prospective, long-term studies are needed to optimize patient selection, evaluate developmental safety and determine sustainable outcomes.

## 1. Introduction

Childhood and adolescent obesity represent a major global public health challenge, with prevalence rising sharply in recent decades [1]. In the United States, adolescent obesity has quadrupled over the past 30 years and recent Center for Disease Control (CDC) data (2021–2023) indicate that approximately one in five individuals aged 2–19 years (21.1%) currently has obesity. Prevalence among children aged 6–11 years has reached 20.7%: nearly five times higher than in the 1960s [2,3]. This upward trend parallels a growing burden of metabolic and cardiovascular comorbidities, including dyslipidemia, hypertension, type 2 diabetes mellitus (T2DM), non-alcoholic steatohepatitis, obstructive sleep apnea (OSA) and musculoskeletal complications [4,5]. A substantial proportion of affected youth already display at least one cardiovascular risk factor and obesity frequently tracks into adulthood, markedly increasing long-term morbidity. Pediatric obesity arises from a multifactorial interplay of behavioral, genetic and environmental influences [6]. No single therapy is universally effective [7,8,9]. This comprehensive review was conducted following a structured search approach to capture the relevant literature on surgical and endoscopic interventions for pediatric and adolescent obesity.

Relevant Section

First-line management typically involves multicomponent behavioral interventions combining nutritional counseling, physical activity enhancement, psychological support and family involvement [10,11]. However, many adolescents with moderate-to-severe obesity do not achieve adequate weight reduction with lifestyle therapy alone, necessitating the consideration of more intensive interventions [5,12,13].

Bariatric surgery, primarily Sleeve Gastrectomy (SG) or Roux-en-Y gastric bypass (RYGB), is generally reserved for adolescents with severe obesity (BMI > 40 kg/m^2^ or >35 kg/m^2^ with major comorbidities) and has demonstrated substantial and durable benefits [14,15]. More recently, minimally invasive endoscopic interventions such as intragastric balloons (IGBs) and endoscopic sleeve gastroplasty (ESG) have emerged as potential intermediate options for adolescents with moderate-to-severe obesity, particularly those who are ineligible or unwilling to undergo surgery [16].

Given that severe adolescent obesity may reduce life expectancy by 5–20 years, there remains an urgent need to expand effective, scalable and less invasive therapeutic options [17].

### 1.1. Behavioral Treatment

Current national and international guidelines consistently identify behavioral treatment as the cornerstone of pediatric obesity management [18,19]. Lifestyle-based interventions should be initiated early and delivered as structured, ongoing care, rather than delayed or passive management. Behavioral treatment relies on a multicomponent approach, including nutrition education, promotion of regular physical activity and reduction in sedentary behaviors, with active family involvement [19]. Specifically, nutrition-related behavioral interventions focus on establishing regular meal patterns, improving dietary quality, reducing the consumption of energy-dense and nutrient-poor foods, and limiting sugar-sweetened beverages, while avoiding overly restrictive dietary prescriptions. Physical activity interventions emphasize increasing daily moderate-to-vigorous activity and incorporating movement into everyday routines, favoring enjoyable and developmentally appropriate activities over structured exercise alone. In parallel, targeted strategies aim to reduce sedentary behaviors, particularly screen time, through goal setting, environmental modification and parental modeling [18]. A central component of effective behavioral treatment is the family-based approach, in which parents and caregivers are actively involved in behavior change. Caregivers are supported in modeling healthy behaviors, establishing consistent routines and creating home environments that facilitate healthy choices. Behavioral techniques such as goal setting, self-monitoring, problem solving and positive reinforcement are commonly employed to enhance adherence to and support sustained lifestyle change [18,20]. These interventions aim to promote sustainable behavior changes, rather than short-term weight loss. In younger children, weight stabilization is often the primary goal, to allow for BMI normalization through linear growth. The evidence supports a clear association between treatment effectiveness and intervention intensity and continuity, as highlighted in intensive health behavior and lifestyle treatment models [18]. The current guidelines emphasize the use of a non-stigmatizing, patient-centered approach that integrates behavioral strategies and psychological support to improve adherence and long-term outcomes [18,19].

### 1.2. Bariatric Surgery

Metabolic and bariatric surgery (MBS) has evolved from primarily weight-reduction procedures to interventions that also modulate metabolic and hormonal pathways, providing both sustained weight loss and improvement of obesity-related comorbidities. Originally developed in adults, these techniques have been adapted to carefully selected adolescents with severe obesity, reflecting the recognition of obesity as a chronic and progressive disease. It remains the most effective treatment for adolescents with severe obesity who do not respond to behavioral or pharmacological interventions [21]. The principal surgical procedures in pediatric practice include SG, RYGB and adjustable gastric banding (AGB), used less frequently [14].

SG was first performed in adolescents in the early 2000s and rapidly became the predominant bariatric procedure, due to its favorable balance of efficacy and safety, and currently accounts for approximately 80% of pediatric bariatric surgeries. The technique involves longitudinal gastric resection with preservation of intestinal continuity, resulting in gastric volume reduction and hormonal modulation. In pediatric patients, SG is often preferred because it avoids intestinal bypass, thereby reducing the risk of malabsorption, while still achieving substantial weight loss and improvement in obesity-related comorbidities. However, long-term nutritional monitoring remains mandatory and postoperative gastroesophageal reflux symptoms may occur in selected patients [11,22]. RYGB has been used in the pediatric population since the early 2000s and has also demonstrated significant effectiveness in adolescents with severe obesity. By combining restrictive and malabsorptive mechanisms, RYGB may offer additional metabolic benefits and superior control of gastroesophageal reflux disease in some patients. Nevertheless, it carries a higher risk of micronutrient deficiencies and requires strict adherence to lifelong supplementation and follow-up [6,7,21]. AGB, which was introduced around 200 and was initially adopted due to its reversibility, has progressively declined in pediatric practice due to less favorable long-term outcomes and device-related complications, including slippage, erosion and need for reoperation [5]. Overall, SG and RYGB are effective and relatively safe options for severe adolescent obesity, whereas AGB is now rarely indicated [23]. Nonetheless, the possibility of nutritional deficiencies, long-term complications and challenges with postoperative adherence underscores the need for careful multidisciplinary management [23]. Importantly, pediatric bariatric surgery requires comprehensive multidisciplinary evaluation. Assessment of physical and psychological maturity, family support and the ability to adhere to long-term follow-up are essential prerequisites. When performed in experienced centers within structured programs, metabolic and bariatric surgery represents a safe and effective therapeutic option for carefully selected adolescents.

### 1.3. Endoscopic Sleeve Gastroplasty (ESG)

ESG is an emerging minimally invasive endobariatric technique in which full-thickness endoscopic suturing remodels and reduces gastric volume, promoting early satiety and delayed gastric emptying while preserving normal gastrointestinal anatomy [16,24,25]. Widely adopted in adults during the 2010s, its application in adolescents is more recent. The first documented pediatric cohort included 109 children and adolescents aged 10–21 years [26]. However, the evidence is limited to this single observational cohort, with follow-up restricted to 24 months, and information on long-term safety, pubertal development, bone health and the durability of weight loss remains scarce. ESG should therefore be considered an investigational therapy in adolescents, pending further studies, including randomized controlled trials and long-term evaluations.

### 1.4. Intragastric Balloons (IGBs)

In this context, IGBs have emerged as an adjunctive intervention when lifestyle modification alone is insufficient. In adults, IGBs have been used for over three decades and represent a reversible and non-surgical modality that promotes early satiety and caloric restriction [27]. Adult studies report mean weight losses of 17–18 kg and BMI reductions of 4–9 kg/m^2^, with a favorable safety profile and predominantly mild adverse effects [15]. Serious complications are uncommon [9]. However, the long-term durability of weight loss remains uncertain, as many adults experience partial or complete regain after balloon removal [9]. Despite extensive adult experience, evidence in children and adolescents remains limited. Small pediatric studies conducted since 1998 have reported reductions in BMI and improvements in obesity-related comorbidities, particularly when IGBs are combined with structured behavioral programs [28]. Swallowable devices such as the Obalon system further reduce invasiveness and may improve acceptability in younger patients [9,10]. Nevertheless, challenges persist: adolescents frequently demonstrate low adherence to behavioral interventions and weight loss achieved through conservative approaches is seldom maintained. Psychosocial and environmental factors strongly influence engagement, while predictors of successful weight reduction remain poorly defined. Concerns also persist regarding the potential effects of rapid weight loss on bone mass, although youth with obesity already exhibit reduced bone density and increased fracture risk prior to treatment.

## 2. Materials and Methods

This comprehensive review was conducted using an adapted PRISMA framework to guide the literature search, selection and synthesis of evidence.

### 2.1. Study Design

This review was conducted as a comprehensive review of the literature to summarize the current evidence on surgical and endoscopic interventions for pediatric and adolescent obesity. This review protocol was registered on PROSPERO (Registration number: CRD420261320533). A systematic search of the literature was performed to identify all studies reporting endoscopic or surgical treatments in the pediatric population, up to August 2025. The search was independently conducted by two reviewers (CDA and MO). Records were removed from the selection if both reviewers excluded the articles at the title/abstract screening levels. Disagreements were resolved via a discussion with a third reviewer (ER).

**Figure 1:**
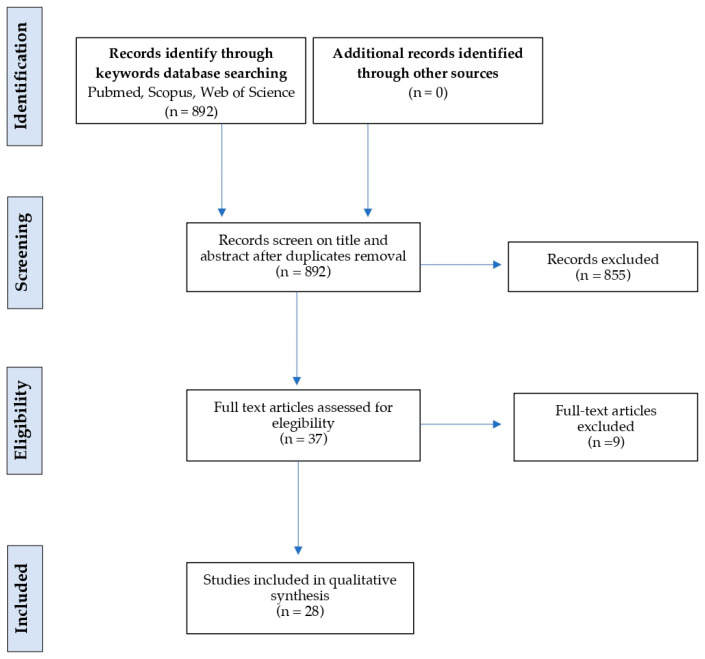
PRISMA flow diagram for study search, selection, inclusion and exclusion.

The following electronic databases were searched: PubMed, Scopus, Web of Science and Cochrane Library. A specific research equation was used for each database, using the following keywords and MeSH terms: pediatric obesity; adolescent obesity; bariatric surgery; and bariatric endoscopy. The inclusion criteria comprised studies that were relevant to the main topic, papers related to children, adolescents or young adults aged ≤ 21 years with overweight or obesity who underwent bariatric surgery (sleeve gastrectomy, Roux-en-Y gastric bypass, adjustable gastric banding) or endoscopic procedures (endoscopic sleeve gastroplasty, intragastric balloons), reporting at least one of the following outcomes: weight loss, BMI reduction, excess weight loss, metabolic or comorbidity outcomes, safety or adverse events, publications as randomized or non-randomized clinical trials, cohort studies, retrospective analyses, pilot studies and case series in English. Manuscripts had to be available in full text and published within the abovementioned timeframe. All retrieved studies were critically reviewed and the data were synthesized. Case reports were only included for intragastric balloon interventions, due to the limited pediatric evidence base. The exclusion criteria were studies involving adults (>21 years), preclinical research (animal or in vitro studies), veterinary investigations, legislative or policy reports, conference abstracts without full-text availability, non-English publications, studies addressing only pharmacological or lifestyle interventions without procedural (surgical or endoscopic) components or articles that were not available in full text. A total of 892 records were identified through searches of PubMed, Scopus, Web of Science and Cochrane Library. After applying the initial filters for English language and age range, we conducted a screening of titles and abstracts. Studies that did not meet our inclusion criteria or articles that were not available in full text were excluded. This process led to the exclusion of 864 records, while the remaining 28 full-text articles were assessed for eligibility and included in the comprehensive review (Figure 1).

### 2.2. Study Selection

All identified records were screened independently by two reviewers, based on their title and abstract. Full texts of potentially relevant articles were subsequently assessed for eligibility. Any discrepancies were resolved through discussion and consensus. The study selection process is summarized in a PRISMA flow diagram (Figure 1).

### 2.3. Data Extraction

Data were extracted using a standardized data collection form. Extracted variables included:

- Study characteristics (author, year, country, study design).
- Patient demographics (age, sex, baseline BMI).
- Intervention details (type of procedure or device, technical characteristics, duration).
- Outcomes (weight loss metrics, BMI changes, metabolic outcomes).
- Follow-up duration.
- Adverse events and safety outcomes.

For intragastric balloon studies, additional details regarding balloon type, filling volume, retention time and anesthesia requirements were recorded.

### 2.4. Data Synthesis

Given the heterogeneity of study designs, populations, interventions and outcome measures, a quantitative meta-analysis was not performed. The results were synthesized narratively and descriptively. The findings were grouped according to intervention type (bariatric surgery, endoscopic sleeve gastroplasty, intragastric balloons) and summarized using text, tables and comparative analysis. Particular attention was given to short-term versus long-term outcomes, as well as to factors influencing treatment durability, including adherence to behavioral and multidisciplinary programs.

### 2.5. Risk of Bias and Evidence Limitations

A formal risk-of-bias assessment was not systematically conducted, due to the predominance of observational studies, small cohorts and heterogeneous methodologies, particularly for endoscopic interventions in pediatric populations. Instead, methodological limitations and potential sources of bias were qualitatively assessed and discussed, including sample size, follow-up duration, study design and completeness of outcome reporting. Although this work was designed as a comprehensive review, a structured methodological quality assessment was performed to enhance interpretability. Cohort and comparative observational studies were assessed using the Newcastle–Ottawa Scale (NOS). Stars (★) were assigned across three domains: selection (maximum 4 stars), comparability (maximum 2 stars) and outcome (maximum 3 stars), for a total possible score of 9 stars. Higher scores indicate a lower risk of bias and better methodological quality. NOS results are summarized in Table 1.

Two reviewers (CDA and MO) carried out the study quality assessment and risk of bias evaluation of the selected articles.

For case series and small pilot studies, where NOS applicability is limited, a qualitative appraisal was conducted, focusing on the clarity of the inclusion criteria, completeness of outcome reporting, adequacy of follow-up and transparency in adverse event reporting. Overall, most studies were observational, single-center and characterized by limited sample sizes and relatively short follow-up, particularly in the field of pediatric endoscopic interventions. These methodological constraints were considered when interpreting the findings.

## 3. Discussion

Accumulating evidence supports MBS as an effective and relatively safe treatment for severe obesity in adolescents and young adults [23]. A recent meta-analysis of 14 studies including 13,994 patients aged ≤ 21 years demonstrated that both SG and RYGB result in significantly greater weight loss and BMI reduction compared with AGB [30]; although RYGB achieved the greatest absolute and relative BMI reduction and it provides superior control of severe GERD compared to SG, it was also associated with a notably higher risk of postoperative complications relative to SG and AGB [14,23,30]. A systematic review and meta-analysis of 37 studies evaluating ~2300 adolescents and young adults (≤22 years) confirmed that laparoscopic SG is associated with substantial BMI reduction and frequent remission of obesity-related comorbidities, including hypertension, T2DM and OSA [14,31]. Data from long-term follow-up (≥5 years) across multiple cohort studies (nearly 5000 patients) show a mean pooled BMI reduction of approximately 13.1 kg/m^2^; the stratified results indicate an average BMI decrease of 15.3 kg/m^2^ for SG and 12.9 kg/m^2^ for RYGB, with AGB yielding more modest reductions (≈7.6 kg/m^2^) [32]. Remission rates for comorbidities (T2DM, dyslipidemia, hypertension, OSA, asthma) were high, ranging from 76 to 92% (specifically, long-term studies report high remission rates for obesity-related comorbidities, with T2DM (90%), dyslipidemia (76.6%), hypertension (80.7%), OSA (80.8%) and asthma (92.5%) [32]. Large registry data also suggest that surgical morbidity and major complication rates in adolescents undergoing SG or RYGB are comparable to those observed in adults, supporting a favorable safety profile under experienced care settings [17]. In summary, SG and RYGB emerge as effective and relatively safe surgical options for adolescent obesity, whereas AGB appears less effective and is increasingly rarely used [23,29]. Nevertheless, given the potential for nutritional deficiencies, long-term complications and variable adherence to follow-up, careful patient selection, multidisciplinary management and lifelong monitoring remain essential [33]. SG and RYGB consistently achieve substantial weight loss and high remission rates of comorbidities; however, most evidence comes from observational cohorts with variable follow-up, single-center studies and selective populations, which may limit the generalizability. AGB appears less effective, but the available data are also constrained by small sample sizes and potential reporting bias.

For ESG, despite growing interest, pediatric evidence remains limited. The most substantial data come from a single monocentric cohort of 109 adolescents with obesity, which reported clinically meaningful and durable weight loss, with a total body weight loss (TBWL) of 14.4% at 6 months, 16.2% at 12 months and 13.7% at 24 months [26]. ESG demonstrated a favorable safety profile, with only mild adverse events, primarily abdominal pain and nausea, occurring in 2–3% of patients and no serious complications or impairment of growth trajectories [16]. Improvements in metabolic parameters were also documented, suggesting additional cardiometabolic benefit. A recent narrative review synthesizing the available data in individuals under 21 years of age confirmed that the pediatric evidence base relies almost exclusively on this single cohort [14]. The review highlighted the proposed mechanisms of ESG, including gastric volume reduction and delayed gastric emptying without disruption of nutrient absorption or appetite-regulating hormones and noted that ESG may provide greater weight loss than lifestyle or pharmacological interventions while carrying fewer risks than conventional bariatric surgery [16]. However, both the cohort study and the narrative review emphasized significant limitations in the current evidence, which is restricted to observational data, lacks randomized pediatric trials and offers limited follow-up beyond 24 months. Data on growth, pubertal development and bone health also remain scarce [14]. Collectively, these gaps underscore the need for larger, multicenter and methodologically robust studies to clarify the long-term role of ESG in the management of pediatric obesity.

With regard to IGB, a total of nine studies were included in this comprehensive review, encompassing case series, retrospective analyses, pilot studies and one multicenter cohort. Studies were conducted across Europe, South America and the United States. They included populations ranging from children to older adolescents (mean ages 12–19 years), with sample sizes varying widely from isolated case reports to cohorts of up to 91 participants. Baseline BMIs across studies ranged from approximately 30 to over 46 kg/m^2^, reflecting populations with obesity to severe obesity. The general characteristics of the studies are reported in Table 2. A variety of IGBs were used, including traditional fluid-filled balloons (ORBERA, Inamed) and newer swallowable devices, such as the Obalon swallowable gastric balloons (SGB) [9,10]. Balloon filling volumes ranged from 400 to 700 mL in fluid-filled systems, while swallowable devices typically relied on fully enclosed gas or water expansion mechanisms, such as the Obalon (17.2 kPa pressure filling) or the SGB (550 mL). The retention time varied from 4 months (SGB) to more than 15 months in one case report, though most conventional IGBs were retrieved at 6 months, as per standard clinical protocols [34]. Anesthetic requirements differed substantially: SGB like Obalon required only pharyngeal anesthesia or mild sedation, whereas traditional endoscopic insertion commonly required moderate sedation or general anesthesia. The inclusion criteria were generally consistent across studies, with most enrolling adolescents who had failed prior lifestyle interventions and met BMI thresholds at or above the 95th percentile or BMI ≥ 30–35 kg/m^2^, with or without comorbidities. The exclusion criteria typically involved endocrine causes of obesity, upper gastrointestinal diseases, psychiatric conditions, eating disorders, pregnancy and contraindications to endoscopic procedures [Table 3]. Overall, the evidence suggests consistent short-term weight loss, favorable safety and considerable variability in long-term outcomes. All included studies consistently reported clinically meaningful short-term reductions in body weight and BMI. Devices such as the Obalon balloon demonstrated significant weight loss at 3 months, with reductions from 95.8 ± 18.4 kg to 83.6 ± 27.1 kg.

Similarly, a study using a saline-filled IGB reported a mean TBWL of 13.05% after 4 months, confirming early efficacy. The SGB study also showed rapid reductions in weight and BMI within the first month, with a weight loss of 7–10% during the initial 3 weeks. By 6 months, most studies documented meaningful reductions in BMI (−2 to −4.3 kg/m^2^), TBWL (12–16%), or excess weight loss (%EWL), often exceeding 20–50%. One retrospective cohort reported a mean %EWL of 56.94%, with nearly half of the participants achieving >50% EWL. Improvements in metabolic markers were also noted, including reductions in fasting insulin and the homeostasis model assessment of insulin resistance (HOMA-IR). However, one small case series (n = 5) described initial weight loss followed by complete rebound by month 6, with BMI percentiles worsening beyond the baseline. This was attributed to poor adherence and compensatory hypercaloric intake, underscoring the considerable variability in outcomes and the central role of behavioral engagement. Only a limited number of studies extended follow-up beyond balloon retrieval. Long-term findings were mixed: one study reported that only two participants maintained weight loss at 24 months, while most regained or exceeded the baseline weight; another documented a weight increase of +9.9 kg at 24 months, despite initial improvement. In contrast, a study using IGB as a bridging therapy before orthopedic surgery reported sustained improvements, with 61.1% of participants maintaining ≥50% EWL. Overall, while short-term outcomes were consistently positive, the long-term durability of weight loss varied substantially and appeared to be highly dependent on adherence to structured, multidisciplinary care programs. Adverse events were common but generally mild across all studies. Frequently reported symptoms included nausea (5–11 cases per study), vomiting, abdominal pain or cramping, transient diarrhea and flatulence, typically occurring during the first week after insertion and resolving spontaneously or with medication. Serious adverse events were rare; however, one case described severe gastric distension with vascular compression due to prolonged balloon retention, highlighting the importance of timely follow-up and retrieval. Across studies, dropout rates were low and several cohorts reported no treatment discontinuations. Data are summarized in Table 4.

Despite the growing array of therapeutic strategies for pediatric obesity, the current evidence base clearly demonstrates that procedural interventions (surgical and endoscopic) play a pivotal role for selected adolescents with moderate-to-severe disease. Bariatric surgery remains the most rigorously studied option, with SG and RYGB consistently achieving substantial and durable weight reduction alongside high remission rates of major cardiometabolic comorbidities in adolescents with severe obesity. Nonetheless, the invasive nature of these procedures, combined with the lifelong need for nutritional surveillance and adherence to follow-up, underscores the importance of careful patient selection and multidisciplinary oversight. ESG represents a promising, less invasive alternative that may help to bridge the therapeutic gap between lifestyle interventions and surgery. Early pediatric data suggest clinically meaningful efficacy with an excellent safety profile. IGBs similarly offer a reversible, short-term benefit, yet post-removal weight trajectories vary widely and appear to be highly dependent on behavioral engagement and structured multidisciplinary care.

The structured methodological assessment further highlighted the limitations of the current evidence base. Most studies were observational, frequently single-center and characterized by limited sample sizes and short follow-up durations. While several cohort studies achieved moderate methodological quality according to the Newcastle–Ottawa Scale, the absence of randomized controlled trials and the reliance on case series for endoscopic interventions reduce the overall strength of the evidence. These factors should be considered when interpreting reported efficacy and safety outcomes, particularly for ESG and IGB in pediatric populations.

## 4. Pediatric-Specific Considerations: Ethics, Consent, Psychosocial Outcomes and Development

Procedural interventions in pediatric obesity present specific ethical and developmental challenges that extend beyond simple clinical efficacy. In this age group, decisions regarding invasive interventions require careful balancing of the potential benefits against the long-term risks, given the limited evidence on safety and durability in pediatric populations. It is widely recognized that treatment decisions should involve not only caregivers but also the adolescent, through an age-appropriate consent and assent process, supported by a multidisciplinary team [35,36]. Equally important are the psychosocial outcomes and quality of life, as severe obesity and related procedures can affect self-esteem, body image and social functioning. While some studies report psychological improvements post-intervention, outcomes are variable and highlight the need for ongoing psychosocial support. Finally, in growing children and adolescents, monitoring pubertal development, linear growth and nutritional status is essential, as significant weight loss may impact endocrine maturation and bone health, requiring long-term surveillance.

## 5. Conclusions

In conclusion, these findings highlight the therapeutic potential and the current limitations of procedural approaches in youth. Establishing their precise role within the pediatric obesity treatment continuum will require high-quality prospective and randomized studies, given the predominantly observational nature of the current evidence. Strengthening the quality and consistency of the evidence will be essential to inform clinical guidelines and to support the development of safe, effective and scalable interventions that are capable of altering the lifelong trajectory of obesity-related outcomes.

## 6. Future Directions

Future research should focus on rigorously designed prospective studies with standardized outcomes to ensure the reliability and comparability of findings. It is essential to extend the follow-up into adulthood to capture the long-term effects of interventions, including their impact on growth, pubertal development and bone health. Additionally, direct head-to-head comparisons with modern anti-obesity medications are needed to determine the relative efficacy and safety, while cost-effectiveness analyses will provide crucial information to inform healthcare policy and optimize resource allocation. Collectively, these approaches will help build a robust evidence base for effective, sustainable and clinically relevant obesity management strategies. Given the escalating burden of pediatric obesity and its profound lifelong consequences, expanding safe and effective intermediate therapies remains an urgent clinical priority.

## Figures and Tables

**Table 1:** Methodological quality assessment of included studies, using the Newcastle–Ottawa Scale (NOS).

Study	Selection (Max 4)	Comparability (Max 2)	Outcome (Max 3)	Total (Max 9)	Estimated Quality
Nocca et al., 2014 [11]	★★★	★	★★	6/9	Moderate
Alqahtani et al., 2019 [26]	★★★★	★	★★★	8/9	Good
Akinkuotu et al., 2019 [13]	★★★	★	★★	6/9	Moderate
Castellani et al., 2017 [29]	★★★	★	★★	5/9	Moderate–Low
Pelizzo et al., 2025 [16]	★★★	★	★★	5/9	Moderate–Low

**Table 2:** General characteristics of the included studies.

	First Author	Year	Journal	Country/Study Center	Study Design	Study Period	Patients’ Age	N° Patients	Baseline BMI
1	F. De Peppo	2016	Thieme	Italy	Case series	Jul 2013–May 2015	12.6 ± 2.3 y	17	35.27 ± 5.89
2	R. J. Fittipaldi-Fernandez	2017	Crossmark	Brazil	Retrospective longitudinal	Jan 2010–Dec 2016	14–19 y	27 (4 M, 23 F)	37.04 ± 6.29 (29.71–56.59)
3	P. Sachdev	2018	International Journal of Obesity	South Yorkshire	Pilot study	2 years	Adolescents	12 (5 M, 7 F)	46.4 ± 5.6 kg/m^2^
4	C. Oyola	2024	IFSO	Chile, Spain, Italy	Single-arm, multicenter, retrospective	Apr 2018–Sep 2022	16.4 ± 0.77 y	91 (22 M, 69 F)	35.60 ± 5.59 kg/m^2^
5	R. Siretskiy	—	The Florida Medical Student Research Publications	Colombia, USA	Case report	2019–2021	17 y	1 F	31
6	L. J. Reece	2017	International Journal of Obesity	—	Non-randomized pilot study	Oct 2012– Jul 2013	Mean 15 y	12 (5 M, 7 F)	—
7	C. T. Pezzo	2017	Nutrire	Brazil	Longitudinal study	Nov 2014–Apr 2015	15.1 ± 0.6 y	10 F	41.3 ± 1.4
8	Y. Vandenplas	1998	European Journal of Gastroenterology & Hepatology	Belgium	Case series	—	14.1 y (range 11.2–17.2)	5 (2 M, 3 F)	147–292% of ideal weight
9	M. H. Soliman	2024	The Egyptian Journal of Surgery	Egypt	Case series	Jan 2021–Dec 2022	12 y (±1.25 SD)	18 (11 M, 7 F)	32.24 ± 1.13 kg/m^2^

**Table 3:** Technical and procedural characteristics of intragastric balloon and patient selection criteria.

	First Author	Type of Balloon	Balloon Volume (Filling)	Balloon Retention Time	Type of Anesthesia	Inclusion Criteria	Exclusion Criteria
1	F. De Peppo	Swallowable Obalon	17.2 kPa	18.61 ± 2.36 weeks	Pharyngeal hypoesthesia with xylocaine spray or deep sedation	BMI > 30 kg/m^2^ with obesity-related diseases (dyslipidemia, OSAS, NASH, GERD) or BMI > 35 kg/m^2^ with/without comorbidities	Hormonal/genetic obesity, upper GI organic diseases, previous GI surgery, anti-inflammatory or anticoagulant therapy
2	R. J. Fittipaldi-Fernandez	IGB	3% saline +10 mL 4% methylene blue (600–700 mL)	6 months	Deep sedation	Adolescents 14–19 y with BMI ≥ 29 kg/m^2^ (>97th percentile) who failed structured clinical programs	Adults, elderly, hormonal/genetic obesity, alcohol/drug abuse, malignancy, pregnancy
3	P. Sachdev	IGB	500 mL saline + methylene blue	6 months	General anesthesia	—	—
4	C. Oyola	Swallowable gastric balloon (SGB)	550 mL distilled water	4 months	Sedation	Age 15–17 y, non-responsive to behavioral/dietary treatments, BMI ≥ 27 kg/m^2^, ability to assent and comply with follow-up	Swallowing difficulty, GI obstruction risk, gastric perforation, GI bleeding, psychiatric illness, eating disorders, pancreatitis, severe respiratory disease
5	R. Siretskiy	IGB	—	15 months	—	—	—
6	L. J. Reece	ORBERA IGB	500 mL	6 months	General anesthesia	—	—
7	C. T. Pezzo	IGB	400 mL saline + 1% methylene blue	6 months	Sedation with midazolam (5–10 mg) and fentanyl (25–50 mcg)	—	Hormonal/genetic obesity, upper GI disease, diabetes (type 1 or 2), prior GI surgery, corticosteroids, anti-inflammatory or anticoagulant therapy; no alcohol/smoking
8	Y. Vandenplas	IGB	500–700 mL saline	6 months	—	—	—
9	M. H. Soliman	IGB	550–600 mL saline + 10 mL 4% methylene blue	6–9 months	General anesthesia	Age 9–16 y, BMI ≥ 95th percentile, failure of dietary weight-loss attempts	Endocrine or upper GI disease; history of weight-control surgery

IGB: intragastric balloon; OSAS: obstructive sleep apnea syndrome; NASH: non-alcoholic steatohepatitis; GERD: gastroesophageal reflux disease; GI: Gastrointestinal; and BMI: body mass index.

**Table 4:** Clinical outcomes, follow-up duration and safety of procedural interventions in pediatric and adolescent obesity.

	First Author	Primary Outcomes Measured	Follow-Up Duration	Results (Summary)	Adverse Events	Severity	Drop-Out Rate
1	F. De Peppo	Weight: 95.8 ± 18.4 → 83.6 ± 27.1 (*p* < 0.05); BMI: 35.27 ± 5.89 → 32.25 ± 7.1 (*p* > 0.05); Excess Weight: 36.2 ± 15.9 →29.4 ± 18.3 (*p* = 0.14); %EWL: 20.1 ± 9.8; WC: 109 ± 12.3 → 99 ± 10.5 (*p* < 0.05)	15 day GPO, monthly until removal	Significant short-term WL; device easy to administer; spontaneous deflation/expulsion in some cases	5 epigastric pain/cramping; 2 epigastric pain; 1 vomiting; 5 nausea; 12 required medication; 1 hematemetic vomiting	Mild–Severe	2
2	R. J. Fittipaldi-Fernandez	WL: 15.99 kg; %TWL: 16.35% (25.92% <10%); %EWL: 56.94% (15% <20%, 37% 20–50%, 48% >50%); 11 patients reached ≤97th BMI percentile	15, 30, 60, 90, 120, 150 days	IGB safe/effective for adolescent obesity; adherence strongly correlated with WL outcomes	—	—	—
3	P. Sachdev	WL 6 months: 7.0 kg (*p* = 0.005); BMI −2.53 kg/m^2^; BMI SDS −0.2 SD (*p* = 0.002); only 2 maintained WL at 24 months	2 years	Effective and well-tolerated short-term; long-term benefits not sustained	11 nausea/vomiting/abdominal discomfort (first week); 1 diarrhea	Mild	2
4	C. Oyola	After 4 months: Weight 86.37 ± 18.83 kg; BMI 30.86 ± 5.16; %TBWL 13.05 ± 7.64	1–4 months (app-based follow-up)	SGB gives safe and effective short-term WL	9 nausea/vomiting; 1 abdominal pain; 1 flatulence	Mild	—
5	R. Siretskiy	BMI 23 (case report)	—	Highlights need for awareness of complications and follow-up in pediatric IGB	Gastric distention; splenic/SMV compression	Severe	—
6	L. J. Reece	12 months: WL 3.05 ± 14.69 kg; BMI Z −0.2 SD at 6 months (not sustained); 24 months: +9.9 ± 1.21 kg	6, 12, 24 months	Safe and well-tolerated; short-term benefits; mixed long-term outcomes	Sickness, diarrhea	Mild	2
7	C. T. Pezzo	First week: BMI −1.74 ± 0.46; WL −6.46 ± 1.52; 6 months: BMI −4.29 ± 1.04; WL −12.9 ± 3.08; %WL day 180: −14.4 ± 2.7; metabolic improvements	Weekly ×2 months, bi-weekly months 3–4, monthly months 5–6	Significant BMI and metabolic improvements with few AEs	5 epigastric pain; 5 nausea; 2 vomiting	Mild	0
8	Y. Vandenplas	First 3 months: WL in all participants; BMI% ↓ non-significantly (*p* = 0.07); at 6 months BMI% > baseline	Every 2–4 weeks until removal	Ineffective long-term; WL reversed by month 6; poor adherence/motivation	3 nausea	Mild	0
9	M. H. Soliman	Mean BMI 25.1 ± 0.93; %EWL 51.54%; TWL 17.17 kg; 61% ≥50% EWL; highest 66.2%; lowest 40%	1 week post-placement, then monthly	IGB effective as bridging therapy before orthopedic surgery	5 nausea/vomiting related to meals	Mild	0

IGB: intragastric balloon; SGB: swallowable gastric balloon; WL: weight loss, EWL: excess weight loss, %EWL: percentage of excess weight loss; TBWL: total body weight loss; %TBWL: percentage of total body weight loss; TWL: total weight loss; ↓: reduction; BMI: body mass index.

## Data Availability

This study is a comprehensive review based on the previously published literature. No new data were created or analyzed and therefore data sharing is not applicable.

## Abbreviations

The following abbreviations are used in this manuscript:

AAP	American Academy of Pediatrics
AGB	Adjustable Gastric Banding
BMI	Body Mass Index
BMI SDS	Body Mass Index Standard Deviation Score
CDC	Centers for Disease Control and Prevention
EWL	Excess Weight Loss
%EWL	Percentage of Excess Weight Loss
ESG	Endoscopic Sleeve Gastroplasty
GERD	Gastroesophageal Reflux Disease
GI	Gastrointestinal
HOMA-IR	Homeostasis Model Assessment of Insulin Resistance
IGB	Intragastric Balloon
IGBs	Intragastric Balloons
MBS	Metabolic and Bariatric Surgery
NASH	Non-Alcoholic Steatohepatitis
OSA/OSAS	Obstructive Sleep Apnea/Obstructive Sleep Apnea Syndrome
PRISMA	Preferred Reporting Items for Systematic Reviews and Meta-Analyses
RYGB	Roux-en-Y Gastric Bypass
SGB	Swallowable Gastric Balloon
SG	Sleeve Gastrectomy
SIEDP	Società Italiana di Endocrinologia e Diabetologia Pediatrica
SIP	Società Italiana di Pediatria
TBWL	Total Body Weight Loss
%TBWL	Percentage of Total Body Weight Loss
TWL	Total Weight Loss
T2DM	Type 2 Diabetes Mellitus
WC	Waist Circumference
